# Multi-Organizational Insights Into Radiological Safety Standards in High-Dose Rate Brachytherapy

**DOI:** 10.7759/cureus.65895

**Published:** 2024-07-31

**Authors:** Anurag Luharia, Shweta B Dahake, Ashish Uke, Gaurav V Mishra, Chanrashekhar Mahakalkar

**Affiliations:** 1 Medical Physics, Jawaharlal Nehru Medical College, Datta Meghe Institute of Higher Education and Research, Wardha, IND; 2 Radiation Oncology, Jawaharlal Nehru Medical College, Datta Meghe Institute of Higher Education and Research, Wardha, IND; 3 Radiodiagnosis, Jawaharlal Nehru Medical College, Datta Meghe Institute of Higher Education and Research, Wardha, IND; 4 General Surgery, Jawaharlal Nehru Medical College, Datta Meghe Institute of Higher Education and Research, Wardha, IND

**Keywords:** ncrp, aapm, iaea, sealed radioactive source safety, radiation protection, radiation safety, brachytherapy

## Abstract

The review provides an extensive study of regulations and recommendations set forth by organizations worldwide in the domain of high-dose rate (HDR) brachytherapy for the prevention and mitigation of radiation hazards. The relevant reports and publications by the International Commission on Radiological Protection (ICRP), International Atomic Energy Agency (IAEA), American Association of Physicists in Medicine (AAPM), United States (US) Nuclear Regulatory Commission (NRC), and Atomic Energy Regulatory Board (AERB) were accessed, and necessary information was compiled to clarify and understand concepts, similarities, and differences in safety standards concerning to the topic. The regulations and guidance are categorized under three major components of safety, namely layout, equipment, and source. Layout category accesses structure, design, layout, and survey. The equipment category summarizes the requirements of equipment, installation, commissioning, quality assurance (QA) and performance, safety precautions and preparedness, safety procedures, and instructions. The source category includes requirements for sealed source possession and use, calibration, categorization, certification, licensing, QA tests, and security. IAEA gives inclusive guidance on radiation protection and regulatory requirements, forming the basis of reference for other organizations worldwide. AERB regulates the radiation facilities in India; therefore, most set-ups follow their safety standards and instructions.

## Introduction and background

Brachytherapy uses sealed radioactive sources to deliver radiation internally or beside the tumor. Brachytherapy treatments began shortly after the discovery of radium. Low, medium, and high-dose rates were used in the past, but high is the most widely used now. Different cases of concern and radiation mishaps involving the safety of medical personnel increased with the increase of high-dose rate (HDR) brachytherapy procedures and have changed radiation protection systems, safety procedures, and management [1]. The International Commission on Radiological Protection (ICRP) was founded in 1928 at the second International Congress of Radiology. The organization provides concepts, definitions, and recommendations for ionizing radiation quantities and units based on biological impacts to safeguard people, animals, and the environment. ICRP recommendations, published in the Annals of ICRP, underpin radiation protection policy, regulations, guidelines, and practices worldwide [2]. The National Commission on Radiation Protection and Measurements (NCRP) was founded simultaneously in the United States (US) for the radiation protection of workers and the public [3].

The International Atomic Energy Agency (IAEA), a United Nations (UN) agency founded in 1957, advises on safe and peaceful nuclear energy use. The American Association of Physicists in Medicine (AAPM) was unanimously approved in 1958 as a scientific, educational, and professional medical physics organization. The AAPM issues radiation safety and efficiency guidelines for medicine. The US Nuclear Regulatory Commission (NRC) was founded in 1975 after the Energy Reorganisation Act 1974 [4,5]. It produces yearly references, regulatory programs, legislation, and regulations to ensure nuclear radiation safety. The President of India established the Atomic Energy Regulatory Board (AERB) in 1983 under the Atomic Energy Act of 1962. In India, the AERB provides safety codes and safety guides and oversees medical radiation facility installation and operation [6].

Every safety advice, guideline, and report aims to enhance the safety of radiation consumption for patients, personnel, and the public. The safety laws and requirements are established based on previous accidents or evidence obtained through various methodologies, while the recommendations are suggested as the furthest level of safety procedures. The organizations using sealed radioactive sources in medicine must abide by the regulatory body and the established compliances according to the territory, state, or country and the regulatory authority [4,6,7]. This review aims to collect necessary information from various documents and compile them under one title to clarify and understand the concepts, similarities, and differences in safety standards per different organizations regarding HDR brachytherapy. It also attempts to organize the elements of safe usage and examine safety standards to draft the specialties of the approaches taken by different organizations to interpret an overall picture of radiation safety standards for HDR brachytherapy at a medical radiation facility.

## Review

Methodology

Four reviewers, including two medical physicists, one radiation oncologist, and one radiologist, independently searched the websites and publications of the organizations to maximally accumulate the recommendations/guides/reports/standards relevant to the keywords. The websites and databases were searched for more than four weeks with keywords like radiation safety, brachytherapy, installation and commissioning of brachytherapy facility, radiation protection, sealed radioactive sources, source safety and source security, and public safety. Two reviewers independently browsed each document downloaded from the searching process. The documents discussing the organizational framework, referring to sealed radioactive sources, and discussing the implementation of safety standards in remote afterloading (RAL) HDR brachytherapy for medical use were included in the study. The reports/documents addressing the governing bodies about establishing regulatory infrastructure were excluded from the study.

Results

The review covers HDR brachytherapy facility installation and operation safety, categorizing data into layout, equipment, and source. Layout is the location and infrastructure for starting the facility, including design, construction, shielding, etc. The equipment category evaluates the RAL HDR unit's requirements, approvals, design, installation, commissioning, and quality assurance (QA) tests. The source category includes sealed source possession and use, calibration, categorization, certification, licensing, QA tests, and security.

In 2014, IAEA Safety Standards Series No. General Safety Requirements Part 3, Radiation Protection and Safety of Radiation Sources: International Basic Safety Standards replaced the 1996 publication. It covers mainly the safety and protection standards and the roles of different people at different levels. The guideline covers planned, emergency, and existing exposures. Most HDR brachytherapy scenarios are planned exposure situations for the patient and, to a certain extent, are expected for the personnel. Emergency exposure situations, such as a malevolent act, accident, or natural disaster, are rare but require immediate intervention. Existing exposure applies to residual radioactive material from earlier operations that were not regulated or that exists after an emergency exposure event. Even with strict definitions, there may be scenarios when the exposure is difficult to classify as planned, emergency, or existent. The General Safety Requirements (GSR) recommends classifying such situations by assessing practical implementation and necessary actions [8].

IAEA Specific Safety Guide, Radiation Protection and Safety in Medical Uses of Ionizing Radiation, Specific Safety Guide (SSG) No. SSG-46, a successor to IAEA Safety Report Series No. 38, discusses radiation protection and safety in medical uses of ionizing radiation in sections 2 and 5 [9]. IAEA Safety Report Series No. 47, Radiation Protection in the Design of Radiotherapy Facilities, covers shielding materials, calculation methods, and radiation surveys. This review does not include shielding calculations; readers should consult the report series for a diagrammatic explanation [10]. AAPM Medical Physics Practice Guidelines (MPPG) 13.a: HDR brachytherapy, section A, details the practical implementation of the pre-established regulations for brachytherapy application and distinguishes between mandatory and recommended actions [11].

In 1972, NCRP published Report No. 40, Protection Against Radiation from Brachytherapy Sources, which addressed personnel safety in terms of maximum permissible dose (MPD) equivalent and other equipment facilities, safety precautions in clinical application, contamination and accidents, and general working conditions for low-dose rate (LDR) and HDR sources. Since this study was issued during the development of RAL units in brachytherapy, little is written regarding the RAL method and its specific recommendations [12]. Sections 3, 4, and 7 of NCRP Report No. 49, Structural Shielding Design and Evaluation for Medical Use of Rays and Gamma Rays of Energies up to 10 MeV, were cited for shielding materials, details, and general brachytherapy safety. In NCRP Report No. 40, barrier thickness calculation for brachytherapy sources is explained. This report covers treatment vault design and construction [13]. In April 2019, NCRP Report 182, Radiation Safety of Sealed Radioactive Sources, updated 1998 guide NCRP Report 127, Operational Radiation Safety Program, on radioactive source safety, was published, which covers source safety from sealed source, fabrication, manufacturing, acquisition, safety during use, testing, disposal, and emergency preparedness. The appendix section of Report 182 clarifies source inventory procedures and license requirements [14].

The AERB Safety Guide (SG), Security of Radioactive Sources in Radiation Facilities, Guide No. AERB/RF-RS/SG-1 covers security concepts, source categorization, and security procedures for Indian radiation facilities. Source security measures are compiled in the report under subheadings of technical and physical protective measures for LDR, HDR, and other radiation facilities. The guide specifies source processing and storage based on security, usage, and customary practice [15]. The AERB Safety Code (SC), Radiation Therapy Sources, Equipment and Installations, Code No. AERB/RF-MED/SC-1 (Rev.1) concentrates on modern radiation therapy sources and applications, listing requirements under titles like safety specifications for radioactive sources, radiation therapy equipment and protective devices (teletherapy, brachytherapy, new equipment, etc.), radiation therapy installation, operational safety, QA, and management of radiation emergency [16].

The US Nuclear Regulatory Commission Regulations Title 10, Code of Federal Regulations (CFR), covers nuclear energy applications. Part 35 covers the guidelines for medical use of byproducts (radioactive material). It discusses the specifications for certain types of brachytherapy equipment, their required specifications, and the handling and regulations for mobile radiation facilities. Parts 20 and 36 cover radiation protection standards, licenses, and safety [17].

Table 1 briefly elaborates on the layout recommendations made by various organizations viz. IAEA, NCRP, AERB, AAPM, and NRC. In column 1, sections 2-4 of the GSR by IAEA are compiled, which elaborate on safety considerations for the planned exposure situation, including any radiation installation emergency [8]. Individual medical radiation facility safety values by IAEA SSG 46 [9] and suggestions regarding the layout and construction from SRS 47 are also combined [10]. Recommendations from the NCRP Reports 40, 49, and 182 on vault shielding, design, and construction are summarized in column 2 [12-14]. In column 3, the compilation of recommendations by AERB SGs and SCs is provided [15,16]. In column 4, the suggestions regarding the layout construction and design from MPPG 13.a are compiled [11]. A brief mention of layout recommendations from NRC CFR 10 has been covered in column 5 [17].

**Table 1:** Layout recommendations by organization IAEA: International Atomic Energy Agency; NCRP: National Commission on Radiation Protection and Measurements; AERB: Atomic Energy Regulatory Board; AAPM: American Association of Physicists in Medicine; NRC: Nuclear Regulatory Commission; EBRT: External beam radiotherapy, LINAC: Linear accelerator; HDR: High-dose rate; MPD: Maximum permissible dose; CFR: Code of Federal Regulations; RAL: Remote afterloading; US: United States.

	IAEA	NCRP	AERB	AAPM	NRC
Requirements for an RAL installation	The location of a radioactive storage facility should consider factors such as safe management, occupational and public exposure, and engineering design feasibility. Registrants and licensees should also consider features that might affect the facility's protection, the amount of radioactivity to be stored, and the feasibility of off-site protective actions. The location should provide easy access to inpatient and outpatient services and simplify radiation protection requirements. Future expansion should consider replacement with higher energy units and increased workload. The location should restrict public access to source storage rooms. The department should be situated away from high occupancy areas and on the periphery of the hospital complex [8-10].	The location of a therapy room should consider operational efficiency, future expansion feasibility, and initial therapy installation costs. Floor rooms should be considered for costs of excavation, watertight sealing, wall shielding, and additional structural support. Construction costs become secondary if the location is near adjunct facilities, provides easy access for outpatients and in-patients, and integrates all therapeutic radiological services. Future room expansion should consider the cost and inconvenience of larger equipment and increased workload [12-14].	The installation of radiation therapy equipment in a hospital must be approved by a competent authority and follow the Atomic Energy (Radiation Protection) Rules, 2004, and AERB Safety Codes. The user institute must obtain regulatory consent from AERB as per the AERB Safety Guide for Consenting process for radiation facilities. No regulatory clearance is issued unless the user complies with these requirements [15-16].	-	-
Structure	A radiation therapy facility consists of various components such as a reception area, clinical consulting areas, EBRT room, brachytherapy room, imaging, and treatment planning room. The structure should consider workload, staff, and patient flow. Treatment rooms should be surrounded by low occupancy rooms and have structural shielding. Access to the room should be through a well-shielded door or maze, and an open access conduit for dosimetry equipment cables should run at an angle through the barrier [8,9].	The size of a treatment room is guided by the choice of equipment and the type of equipment, patient type, and the use of special equipment for research and teaching. Construction costs must be considered against future convenience or the need for additional equipment. Access to the room is provided by a shielded door, with adequate shielding and auxiliary means for power failure or mechanical breakdown. Doorways, elevators, and mazes must be adequate for equipment delivery. Access to replacement sources in large shipping containers should be considered [12-14].	The layout of a brachytherapy facility should be functionally efficient, allowing easy and safe procedures. The doors, passages, and turnings should allow easy transport of equipment, source transfer flask, and patients. The entry to the treatment room should be indirect to minimize shielding requirements. Approval of the room layout plan requires consultation with experts, including medical physicists, radiation oncologists, architects, and the unit supplier. After finalization, the application must be submitted with PDF files of the drawings as an attachment [15,16].	Shared treatment vaults with EBRT or simulators may eliminate the need for brachytherapy room shielding, but interlocks are required to prevent accidental use of other machines such as LINAC or simulator when the treatment is going on [11].	Two physical controls should be present to secure Mobile Category 2 material when not in direct control of the licensee from unauthorized removal and should have constant surveillance [17].
Design	The design of a treatment room should include physical signage, designated hazardous areas, an air conditioning system. The entrance should be through a shielded door or maze or a combination of both. The room should be monitored by operators at all times, with a last person out button in place to ensure staff leave before treatment begins. Emergency off switches should be placed inside the room, on the control panel, and on the equipment itself. The maze should be as long and small as possible, with a barrier in the form of a normal door to discourage entry. Ducts and conduits should be shielded between the treatment room and outside, with no duct larger than 30 mm piercing the primary shielding should be present [8-10].	For therapy installations above 150 kV, consider a series-connected interlock system at the access door. Place warning signs and devices at appropriate locations, such as a red warning signal light on the control panel and near brachytherapy room entrances. Post a warning sign in "Radiation Area" for areas where exposure could exceed 5 mR in an hour or 100 mR in five consecutive days. For gamma-ray beam therapy installations, post-emergency action procedures should be present near the control panel. Include a cleaning sink in storage rooms for intracavitary and interstitial applicators [12-14].	The color of indicator lights in treatment control panels, treatment rooms, and treatment rooms should correspond to the status of the radioactive source(s) at the treatment positions, in transit, ready state, and equipment switched on but not yet ready. A door interlock should be provided for automatic treatment interruption and source return in case of door opening during irradiation. The radiation symbol, specified under Rule 14 of Atomic Energy (Radiation Protection), 2004, should be prominently posted at the treatment room entrance and controlled areas. A legend in Hindi and English indicating radiation hazards and restricted entry should also be posted [15-16].	During the design phase, the patient procedures requiring ancillary devices and equipment should be considered. A wide maze and doorway facilitate gurney transport, while a small shielded enclosure serves as an emergency container and routine storage area [11].	Licensees must disable devices on vehicles or trailers unless site health and safety requirements prohibit it and unless under direct control and constant surveillance [17].
Shielding	Shielding calculations should avoid conservative assumptions to avoid unrealistic overestimates. The design and specification of shielding should be done by a qualified medical physicist or radiation protection expert. The shielding's adequacy should be assessed before clinical use. The amount of shielding depends on the surrounding area, estimated workload, dose rate, required dose limits, and instantaneous dose rates. Lead or steel plates can be used to compensate for displaced shielding material due to ducts. Additional shielding is required for penetrations in shielding walls, depending on the radiation beam's energy, room layout, and duct route. Concrete is the most cost-effective construction material, but higher-density materials may be necessary for space-constrained environments [8,9].	Local shielding is preferred for protection against radiation from brachytherapy sources, as it ensures adequate protection for individuals and radiation-sensitive objects. Low-level radiation counting equipment should be placed in storage rooms or workrooms close to a room. Concrete shielding is more advantageous for high-energy radiation, but structural shielding is generally unnecessary during patient treatment. Storage rooms should be locked to prevent unauthorized access and be under responsible user administrative control [12-14].	The treatment room must have structural shielding for walls, ceilings, and floors to prevent radiation doses from exceeding the limits specified in appendix IV. The shielding should consider patient workload, radiation beam use factor, and nearby occupancy. Service and dosimetry openings must be shielded, with exceptions for non-significant radiation hazards under normal working conditions [15,16].	US regulations on shielding design are vague, but they must meet 10 CFR § 20 radiation exposure requirements. Most LINAC vaults provide adequate shielding for HDR brachytherapy, but a survey for hot spots or defects is necessary. Afterloaders must be stored and secured in the treatment vault to comply with regulations. Patients should be treated on non-radiopaque tables if shared with a simulator room [11].	-
Radiation survey	The facility should be equipped with area monitors and portable survey meters for reference and relative dosimetry for beam characterization and quality control. Surveys should be performed periodically around source storage and HDR brachytherapy, and the dose rate at a treatment room should be monitored. Barrier source applicators should be positioned at the patient's position and exposed without phantom. The radiation survey report should include the type of radiation unit, location, date of survey, person responsible, workload, use factors, occupancy factors, instruments used, results, and conclusions on shielding effectiveness. A floor plan of the treatment facility with survey points should also be provided [8-10].	A radiation protection survey is required for new and existing installations, evaluating occupiable areas near radiation installations to determine if any person is likely to receive more than the applicable MPD. Approval or disapproval of the facility should be based on compliance with NCRP recommendations and relevant regulations. Radiation measurement should be performed when scanning reveals radiation fields of significant levels and using properly calibrated devices. Factors such as calibration, stability, sensitivity, energy dependence, rate dependence, size of sensitive volume, time constant, and directional dependence should be considered. A radiation survey meter should be available for source storage or brachytherapy rooms [12-14].	A radiation zone monitor will be installed near the entrance to telegamma and brachytherapy facilities to continuously monitor radiation levels [15,16].	-	A licensed person must conduct surveys to ensure that the maximum and average radiation levels emanating from the main source holder or safe with the source(s) in the shielded position do not exceed the levels mentioned in the Sealed Source and Device Registry. This requirement is must at the installation of a new source and after repairs to electronic or mechanical components that could by any means expose the source or compromise its radiation safety [17].

Table 2 mainly describes the parameters of the equipment for the above-mentioned organizations. In column 1, the requirements for RAL equipment design, safety, and also therapy-related computers have been included from IAEA GSR Part 3 [8]. From NCRP Report 40, only the essential components regarding equipment parameters and source safety parameters have been included in column 2 [12]. In column 3, the inclusion of equipment requirements as per the AERB SC and guide has been provided [15,16]. In column 4, the acceptance testing, commissioning, and QA regulations as per AAPM MPPG 13.a are given [11]. Column 5 includes a summary of NRC requirements for HDR equipment as per CFR 10 [17].

**Table 2:** Equipment recommendations by organization IAEA: International Atomic Energy Agency; NCRP: National Commission on Radiation Protection and Measurements; AERB: Atomic Energy Regulatory Board; AAPM: American Association of Physicists in Medicine; NRC: Nuclear Regulatory Commission; RAL: Remote afterloading; TPS: Treatment planning system; QA: Quality assurance; NOC: No Objection Certificate; IEC: International Electrotechnical Commission; LMO: Last man out; SGT: Source guide tube; ISO: International Organization for Standardization; HDR: High-dose rate.

	IAEA	NCRP	AERB	AAPM	NRC
Requirements for RAL units	The supplier/employer/licensee must ensure that medical radiological equipment and software meet the standards of the International Electrotechnical Commission, International Organization for Standardization, or national standards adopted by the regulatory body. Licensees are responsible for radiation safety and imposing purchasing specifications that meet international standards. The design of medical radiological equipment should be reproducible, accurate, and predictable, meeting the requirements of GSR Part 3 for operational optimization of patient protection. Radioactive sources should be fail-safe, retracting to a shielded position in power interruptions. Safety systems for radiation therapy equipment should prevent unauthorized use, with a key required for energizing the system and restricting access to authorized staff [8].	Radioactive sources must be transported in a way that does not exceed the maximum permissible dose equivalent or dose limit. Protection should be provided through distance or shielded containers, with containers being appropriately labeled. Factors to consider when designing transport containers include lead as the most practical shielding material, long handles on hand-carried containers, and the need for shielded containers with wheels for large sources. Devices with sealed sources must be classified for their intended use and constructed and tested according to industry and consensus standards. Design specifications for sealed sources should include critical features, sealed source containment, movement, mounting, shutter controls, and tamper-resistant features. Special provisions should be made for portable devices. Sealed source certificates and device registrations should be associated with specific information, and certificate holders may need to justify licensing or control levels [12-14].	This code mandates that radiation therapy sources and equipment must meet design safety specifications. Manufacturers and vendors must obtain design certification from competent authorities before marketing and manufacturing. Local manufacturers must obtain a No Objection Certificate (NOC) or type approval certificate for supply, while vendors must obtain an NOC for import. The equipment must also comply with mechanical, electrical, fire, and environmental safety specifications (IEC 6061-1-1, IEC 6061-1-2, IEC 6061-1-3, IEC 6061-1-4) [15,16].	-	The use of photon emitting after the loader unit is used should be approved in the sealed sources and device registry or in accordance with the safety conditions described in the same. For remote afterloaders, the licensee shall account for all sources before departure from the client’s address of use and check the survey instrument. The copy of permission to use the afterloader and the radioactive material at the client’s address should be retained for three years [17].
Installation	The development of brachytherapy equipment procedures requires collaboration between a medical physicist, radiation therapy professionals, and the facility's radiation protection and quality assurance committees. The applications should be tailored to the specific source or compatible with it [8-10].	The manufacturer must test each sealed source/device model in accordance with regulatory requirements to ensure its integrity during normal use, foreseeable mishaps, handling, maintenance, storage, and transportation [12-14].	The supplier must confirm if the radiotherapy unit is type approved by AERB or issued an NOC to the local supplier. A valid certificate is required for radiotherapy unit sales in the country. AERB issues NOCs for new models imported for the first time [15,16].	-	The installation, replacement and relocation of RAL should be performed by a person authorized to do so. Also, any kind of maintenance, adjustment, and repair of the unit should be done by authorized personnel [17].
Acceptance testing and commissioning	Acceptance testing ensures equipment and software compatibility with other equipment, ensuring compliance with the manufacturer's technical specifications and safety requirements from recognized standards. During commissioning, medical physicists identify, measure, and compile data for clinical use, validate the data, define quantities and measures, and set baseline values for periodic quality control tests. This process ensures equipment and software compatibility with other equipment with interfaces [9,10].	-	The accuracy regarding the source positioning, sequence, and electronic timer, linearity, and reproducibility must be checked at commissioning and periodically. The dose-effect due to transit or end dose on dose delivery must also be verified. A battery-powered backup power supply system must be included in case of unplanned power failure. The applicator must position the radiation source(s) correctly and ensure that any structures used for attenuation of radiation, such as bladder and rectal shields, have not been shifted by radiography during initial use or following malfunctions and that dummy and active sources are coincident. The evaluation includes anatomical data input, TPS beam definition, TPS errors and inaccuracies, and TPS functional capabilities and computational paths [15,16].	Tests for RAL include source strength measurement, positioning accuracy, backup battery, timer accuracy, electrical interlocks, last person out switch, treatment interrupt button, source out indicators, emergency response kit, audio/visual systems, independent radiation room monitor, calibrated survey meter, console computer date and time, decayed source strength, catheter misconnect/channel/turret check, and TPS to console software communication. For the commissioning of applicators and guide tubes, autoradiography should be performed followed by geometric integrity of applicator, source path verification, offsets, matching between solid applicator library and physical applicator, and source positioning inside them. For TPS, source validation and dose calculation should include source modeling, decay, plan normalization, dose calculation grid, point dose calculation, dose display, and dose-volume histogram [11].	A licensee should perform the full calibration measurement of the RAL unit at specified events, which are before first medical use, following a source replacement, reinstallation of the unit, after any repair, at an interval of one-quarter at 75 days for sources whose half-lives are longer than that. The tests include checking for output, source positioning, retraction, timer accuracy, linearity and length, and functions of transfer tubes [17].
Quality assurance	In the case of an RAL unit, if a major repair, modification, or source replacement occurs, treatment cannot proceed until the necessary tests are completed and checked by a medical physicist, confirming equipment safety. Safety checks for applicators should include catheters, couplings, and transfer tubes before and after every treatment [10].	-	Daily and weekly quality assurance (QA) checks are conducted on various aspects of a treatment room, including door interlocks, lights, alarms, console functions, switches, batteries, printers, and more. Weekly checks involve ensuring the accuracy of source and dummy loading, source positioning, calibration, timer function, source guides, connectors, and mechanical integrity of applicators. At every source change calibration, timer function mechanical integrity of applicators, and accuracy of guide tubes have to be examined. Annual QA includes a verification of the source inventory dose calculation algorithm and simulation of emergency conditions [15,16].	Daily QA includes source positioning, timing, electrical interlocks, emergency source retraction buttons, last person's out button, treatment interrupt button, audio/visual systems, emergency response kit, independent radiation room monitor, calibrated survey meter, console computer date and time accuracy, and catheter misconnect/channel/interrupt check and timer linearity for RAL. At the time of source change, strength measurement, positioning accuracy, retraction with backup battery failure, timer accuracy, emergency buttons, LMO, interrupts, source out indicator, computer date and time, TPS to console communication, and decay source strength should be verified. For applicators, on a daily basis, a visual inspection of integrity should be done and the annual length of the applicator and SGT combination should be checked [11].	Quality assurance tests or spot checks for the RAL unit have to be performed by a licensee at regular intervals and should authorize the results from a medical physicist within 15 days. The checks should ensure proper operation of electrical interlocks and source exposure indicator lights at various locations. If any malfunction is observed, the licensee shall lock the control console in the off position and shall not use it until it is repaired. While using a mobile RAL unit, before every use, electrical interlocks, source exposure indicator lights, viewing and intercom system, source transfer tubes, radiation monitors, and position accuracy should be checked [17].
Performance of RAL unit	Medical physicists must perform tests to ensure equipment is operating properly before treating patients. Licensees must maintain records of maintenance for medical radiological equipment after breakdowns or exchanges. Both hardware and software should be operated to ensure satisfactory performance at all times [8,9].	-	-	-	-
Safety precautions/preparedness of RAL	Medical radiological equipment operating consoles must comply with IEC and ISO standards and be translated into local languages. Procedures for afterloading units include observing error messages and emergency indicators, recovering at the console, entering the room with a portable radiation survey meter, observing radiation levels, recovering at the afterloading unit, manual retraction of the source, surveying the patient and afterloader, removing the applicator and placing it in an emergency container, and removing the patient from the vault with redundant survey monitoring. Information should be provided to maintenance personnel and regulatory bodies [9,10].	The source custodian must conduct periodic inventories of all sources, with the hospital radiation protection supervisor checking them at intervals of no more than six months [12-14]. Emergency response procedures for sealed sources are required, and NRC regulatory guidance documents provide general direction for various source types. Emergency instructions should be brief and direct, focusing on dose control, contamination control, and concerns of management and the public [12-14].	The emergency action plan at the radiation department aims to manage any emergency situation and mitigate the consequences, aiming to limit external exposure. The licensee must prepare emergency action plans for all foreseeable emergencies, including radioactive source failure, damage, dislodgement, loss, or theft, and patient death. These procedures should be displayed near the control panel in remote afterloading brachytherapy equipment. The plan should identify personnel handling radiation emergencies, provide initial training and drills, recognize abnormal exposures, maintain appropriate tools, and specify authorities to be contacted during the initial phase, during progress, and at the termination of an emergency. The plan should also include training for recognizing abnormal exposures and formal procedures [15,16].	-	A licensee must control access to treatment rooms through doors at each entrance, equip them with an electrical interlock system, and require individuals to use radiation monitors. They must also construct or equip rooms with viewing and intercom systems. For licensed activities involving sources within a patient's or research subject's body, treatments must allow for quick removal of decoupled or jammed sources. High-dose rate remote afterloader units need an authorized user and medical physicist during the initiation and continuation of treatments to be physically present. If a patient or research subject has a medical emergency or dies, they must notify the radiation safety officer. A licensee must ensure proper operation by conducting a simulated treatment cycle before use [17].
Safety procedure and instructions	For the safety procedures or emergency conditions, prerequisites must have an emergency container, an emergency kit containing long-handled forceps, and applicators, and the staff must be trained for handling the situation. In HDR, the response time is short (in minutes) and hence requires a radiological medical practitioner, a medical physicist, and medical radiation technologist available during all applications [8,9].	If a sealed source is disrupted, trained personnel should be available to assist in decontamination. Untrained individuals should not attempt to examine or clean up spilled radioactive material. For Cobalt-60 and Iridium-192, a traffic control program should be instituted to minimize contamination tracking. Equipment like respirators should be assembled, and vacuum cleaning should be performed before wet mopping or scrubbing. Masking tape can be used for adhesion removal, and damp mopping with a detergent and chelating agent can help remove the remaining contamination [12-14].	Emergency procedures for remote afterloading brachytherapy equipment failure or loss must be established and posted. In case of an accident, care must be taken to save lives, minimize radiation doses, and take necessary remedial steps. The competent authority should be consulted to restore normal conditions. Preventing radiation injury, such as direct contact with unshielded sources, is crucial. If contamination is released, affected areas should be isolated and used only after appropriate decontamination. The affected personnel should be assessed for internal contamination and further remedial measures [15,16].	-	A licensee has to secure the unit, console, and console keys. They should also only authorize people to be present during the treatment, prevent dual operation of devices, and develop, implement, and maintain written procedures for responding to abnormal situations, and a copy of those procedures shall be present at the unit console. The licensee has to provide names and contacts of authorized radiation personnel to be contacted in case of an abnormal function. They must ensure that every personnel goes through vendors' operational and safety training provided by the manufacturer. The safety instructions and drills shall be given at least on an annual basis [17].
Therapy-related computers	The TPS should be designed as such to meet the clinical goals of the radiation therapy facility [8].	-	-	-	The licensee must perform acceptance testing of TPS and therapy-related computers in accordance with national criteria. The tests must include source specific input parameters, accuracy of dose and dwell time, treatment time calculation, accuracy of isodose plots, software used in position determination, and electronic transfer of treatment delivery parameters to the treatment delivery unit from TPS [17].

Table 3 compiles the regulations and recommendations stated for source designing, handling, transportation, safety, and QA given by the above-mentioned organizations. Column 1 includes detailed comments on requirements and regulations for source possession, licensing, storage, and QA as set by IAEA GSR Part 3 [8] and other guidelines from SSG 46 and SRS 47 [9,10]. As per NCRP Reports 40, 41, and 182, all the sealed radioactive source-related recommendations are included in column 2 [12-14]. Column 3 briefs the source storage, remote afterloading, source and zone monitoring requirements, dosimetric concerns for instruments, dose delivery, quality checks, radiation emergency management, and regulatory controls from AERB SG and SC [15,16]. AAPM recommends source recipients as mentioned in column 4 [11]. As per the NRC requirement for medical use of radioactive byproducts, the regulations are compiled in column 5 [17].

**Table 3:** Source recommendations by organizations IAEA: International Atomic Energy Agency; NCRP: National Commission on Radiation Protection and Measurements; AERB: Atomic Energy Regulatory Board; AAPM: American Association of Physicists in Medicine; NRC: Nuclear Regulatory Commission; RAKR: Reference air kerma rate; HDR: High-dose rate; CFR: Code of Federal Regulations; RAL: Remote afterloading; RSO: Radiation Safety Officer.

	IAEA	NCRP	AERB	AAPM	NRC
Supplier for sealed sources or devices	The manufacturer and supplier are responsible for providing well-designed, well-constructed radioactive sources and source housing equipment that meet standards, engineering, performance, and functional specifications. They must also ensure prominent displays, gauges, and important instructions on the operating consoles in user-friendly language. They must also test radioactive sources, make information available on proper installation and use, and optimize protection through shielding and protective devices. Radiation sources should be purchased from suppliers meeting national requirements for such dealings [8,9].	The source capsule material should be resistant to corrosion and damage, and manufacturers must clearly state the intended use and licensing level in design specifications. Sources manufactured under an NRC or Agreement State license must meet specific regulatory performance requirements. Engineering drawings should be prepared for each sealed source, including a construction diagram with dimensions, tolerances, and materials. Design specifications should include radionuclides with maximum allowable quantities, acceptable levels of contaminants, and recommended leak-test frequency [12-14].	-	-	A licensee is allowed to use sealed sources or devices that are manufactured/labeled/packaged/distributed under a license issued under 10 CFR Part 30 and 10 CFR 32.74 equivalent requirements of an Agreement State [17].
Requirement for possession of source	Organizations and individuals are prohibited from handling or performing actions involving radioactive sources outside of compliance with standards, with notification being sufficient if exposures are unlikely to exceed a small fraction [8,9].	Hospitals and clinics with brachytherapy sources should have a custodian under the supervision of a radiation protection supervisor. This custodian must maintain a permanent record of source order, issuance, and return. To obtain licensed sealed sources, individuals must apply for a radioactive material license from the National Radioactive Council or an Agreement State. RSOs must ensure the sealed source is properly inspected upon arrival, added to the institution's inventory, does not exceed license limitations, completes leak testing, and ensures proper user training in safe storage and use [12-14].	For a source and practices associated with the operation of radioactive sources, a license shall be issued. Transportation of radiation therapy sources requires prior approval of the competent authority. Adequate provisions for the security of sources should be made by the employer/licensee during all stages of handling as required by the competent authority [15,16].	The licensee must take possession of the package during source receipt [11].	Licensees of sealed sources or brachytherapy sources must follow the manufacturer's radiation safety instructions and undergo leakage tests before use. Tests should be conducted at time intervals not exceeding six months or at appropriate intervals approved by the Commission or an Agreement State. If the source fails to meet requirements, licensees must withdraw the sealed sources and file a report within five days [17].
Requirement for use of source	Individuals or organizations planning to use a radioactive source must apply to the regulatory body for authorization, either through registration or licensing [8-10].	A radioactive materials license is required for procuring and possessing sealed sources, and all users and staff should receive proper radiation safety training and programmatic control. Employers should ensure that only personnel work with brachytherapy sources after training and provide physical protection, including a locked enclosure in a secure room. Manufacturers should include items that could impact the source's performance and provide clear use limitations in instructions. Environmental limitations, conditions causing degradation or breach of containment, loss during use, handling, storage, or transportation, and extreme operating conditions should be considered [12-14].	A license is an authorization for sources and practices related to brachytherapy operation, registration for radiation installation siting, design, construction, commissioning, and decommissioning, and consent for sealed sources and radioactive-containing equipment for manufacture and supply purposes, as well as for the operation of radiation installations should be checked [15,16].	-	A licensee shall use sealed sources that are RAL-approved and are in accordance with the one provided in the Sealed Source and Device Registry [17].
Calibration of source	Medical physicists must calibrate all sources of medical exposure using internationally or nationally accepted protocols. Calibrations occur at commissioning, before clinical use, after maintenance, and at approved intervals. RAKR or equivalent quantity should be used. All sources used should be individually calibrated, and differences over 5% from the manufacturer's certified RAKR should be investigated [8,9].	Medical therapy sources must be calibrated to determine exposure or absorbed dose rates under specific conditions, with uncertainty clearly stated between the calibration service provider and the user [12-14].	At the specified distance of 1 meter from the brachytherapy source(s), the reference air kerma rate shall be measured to an accuracy of ±5%, with the help of a properly calibrated instrument, and it should be verified annually, or earlier, in conditions like sources changed or suspected to have undergone any damage. Any unexpected deviation in the measurements must be investigated, and appropriate corrective action must be taken before re-using the sources. Also, a report of that shall be sent to the competent authority [15,16].	-	A licensee must determine source output and mathematically correct outputs for physical decay, before first medical use of a brachytherapy source, at intervals consistent with 1% decay [17].
Categorization of source and source security	The classification of sealed sources should adhere to the scheme outlined in Schedule II and the regulatory body's requirements [8].	The HDR brachytherapy sources are classified as Category 2 sources that have an A/D ratio of 10 and <1,000 [12-14].	HDR brachytherapy sources are classified as Category 2 sources, with a Security Level B. To maintain security, formal security plans, critical information security, and background checks on key personnel, response to increased threat perception and availability of emergency response plans are essential [15,16].	-	-
Identification, certification, and licensing	To ensure accurate source identification, HDR sources should be labeled with the International Organization for Standardization symbol and accompanied by a source certificate detailing source strength, quality control tests, and leakage and contamination tests. An inventory of sources should include the radionuclide, location, activity, reference date, serial or batch number, and unique identifier. Sealed sources used for brachytherapy should also have a calibration certificate from the manufacturer [8,9].	The user must be provided with a detailed description of all sources containing radionuclide and their capsules. Regulations dictate markings for sealed sources/devices, which should be durable and legible. This information can be engraved or etched onto the device, or affixed to a metal tag. Labels should be mounted on the device's portion, not on a detector housing or guard barrier. They should be easily visible to users or personnel near the device [12-14].	Encapsulation of sealed sources and containers should include information such as the radionuclide's mass number, source serial number, target element, and manufacturer's name. A source certificate should also provide information on the air kerma rate, dimensional details, tests performed, and the recommended useful life of the source. Physically possible markings should be placed in the container [15,16].	-	-
Source housing integrity	-	To minimize potential exposure, provide adequate shielding in occupied or potentially occupied areas near source storage areas, and protect sealed sources from hazards like damage, fire, and flooding [12-14].	Remote afterloading brachytherapy equipment source housing must maintain source integrity and shielding, with a fail-safe mechanism in the unit and room to prevent personnel exposure and excess dose to patients [15,16].	-	-
Source storage and security	Radioactive sources should be stored securely and not left in the applicator. In case of a failure of the afterloading unit, the radiation therapy facility should have a storage container, remote manipulator, wire cutters, and a radiation monitoring instrument. Security measures should be taken to prevent unauthorized access and detect unauthorized access. For storage sources, a locked container, locked room, access control, and the capability to detect unauthorized access are necessary. For HDR sources, used in a locked room or controlled area, constant surveillance and control of access to the area by unauthorized people are required [8-10].	Brachytherapy sources and applicators should be stored in a protective, locked enclosure to reduce exposure rates. The outer surface should be made of fire-proof materials. Separate compartments should be provided for different source activities. Licensees must maintain records of receipt, transfer, and disposal of sealed sources, with records maintained for at least three years after disposal. The inventory should provide easy access to storage locations, usage dates, leak testing results, and final disposition. Stored sources should minimize exposure to workers and the public and deter loss, theft, or damage [12-14].	The source storage design should provide adequate protection for operating personnel, prevent unauthorized access, and maintain source integrity under normal operations and anticipated accidental conditions. The in-house transport container should have a radiation symbol displayed on its exterior, and temporary storage containers should ensure leakage radiation levels do not exceed the limits [15,16].	-	-
Quality assurance	Registrants and licensees must establish a comprehensive quality assurance program for medical exposures, with a team of medical physicists, radiological medical practitioners, and medical radiation technologists. The program includes measurements of physical parameters of medical radiological equipment, corrective actions if measured values are outside tolerance limits, and periodic checks of calibration and operation conditions. Sealed sources should undergo leak tests before and after use, in accordance with international standards, to detect small amounts of removable contamination. These tests should be conducted on afterloading drive assemblies and transport containers [8-10].	Periodic leak tests on sealed sources are required, with frequency specified by local, state, and federal regulations. If tests reveal 0.005 or more removable contamination, the source is leaking. It has to be removed, sealed, and returned to the supplier or a qualified person for repair or disposal. Typically, leak tests occur at intervals of no more than six months. All packages must be visually inspected for damage and monitored for radiation and contamination levels [12-14].	Source integrity and uniformity must be checked for all radioactive sources before use and re-checked at intervals of six months. Initial procurement includes physical/chemical form, encapsulation, radionuclide distribution, uniformity, location, mean activity, and deviation from mean [15,16].	-	A licensee must ensure the accurate positioning of a brachytherapy source within applicators before its first medical use [17].

Layout

The term layout, or the room layout, refers to the design, dimensions, construction, and shielding of the treatment room or treatment vault of the RAL HDR installation to ensure safety when the machine is ON. The broad term encompasses various construction requirements as guided by organizations, the structure of the room, its design, shielding, and the radiation survey that has to be performed at the beginning and regular intervals during as well as after construction.

Requirements for a RAL Installation

To install a RAL HDR brachytherapy machine, the organization or individual has to start with the necessary setup for a correctly placed and functional room that can and will accommodate the machine, along with the source, and facilitate the delivery of treatment in a way that is safe for patient, personnel, and public. This entails a few mandatory requirements and others that suggest safer practices.

Structure

The facility's structure refers to the floor plan and dimensions of the treatment room or vault. AAPM makes suggestions for the creation of a dedicated treatment room for brachytherapy procedures [11]. NRC Guideline 37.53 of 10 CFR specializes in mobile therapy equipment and the necessary structure requirements [17].

Design

Designing the facility concerns the efficient and effective placement of necessary components of the treatment vault to maximize the setup's functionality and safety. NCRP Report Number 40 also shares the maintenance of sinks [12].

Shielding

The specially designed and constructed barrier attenuating the primary, secondary, and scattered radiations from the source in both ON and OFF states is compelled and needed to protect professionals and the general public.

Radiation Survey

This comprises monitoring the layout/installation after installation of the source, which is being considered in different aspects in different reports.

Requirements for RAL Unit

The defined requirements for an RAL unit apply to the organization or user who intends to acquire and operate an RAL unit and the manufacturer.

Installation

Installing involves purchasing the equipment and placing it in the treatment vault before commissioning it for usage.

Commissioning

After the installation, there is a need to verify the structures, systems, and components of a brachytherapy facility in accordance with design specifications to make the setup functional for use and check the performance according to the set criteria.

Quality Assurance (QA)

Many international and national organizations and regional professional bodies have published detailed guidance on the range of acceptance, commissioning, and quality control tests that should be performed on radiation therapy equipment and software, how they should be performed, applicable tolerances and desired action levels, and recommended frequencies. Due to improper post-repair testing, significant medical exposure occurred. IAEA rules provide RAL and applicator QA. The AERB and AAPM suggest RAL unit and guide tube test frequency and techniques. The NRC document regulates the same and advises licensees to complete and record checks [8,11,15-17].

Equipment

Equipment, in general, refers to the machine or unit used to deliver the radiation dose in the current most widely practiced form of HDR brachytherapy. The equipment is the remote afterloading unit, which contains, stores, and safeguards the source and delivers treatment on an instruction basis from the treatment console itself. This device is solely responsible for minimizing the radiation dose to professionals as the applicators are inserted and connected before treatment delivery. No one except the patient is inside the treatment vault while the source is out of the shielded position. The equipment section will also consider the guide tubes and applicators used in the process.

Performance of RAL Unit

Performance of RAL unit refers to equipment functionality and efforts to maintain optimal performance. This may include other needs, procedures, and actions for a well-functioning and equipped brachytherapy setup. SSG-46 recommends translating operation manuals and comparable instructions into the local language. The operating instructions are used by certified professionals and technicians who may not speak any major world language. To avoid operating faults and assure understanding, the translation needs QA. The same goes for maintenance instructions for service engineers and technicians [9].

Safety Precautions, Safety Procedures, and Instruction

While the RAL HDR unit is operating and functioning daily, certain procedures and precautions must be mandated to maintain safety. These procedures are specific to the actual afterloading unit but generally involve a standard sequence. This needs a well-educated and well-trained staff for everyday and emergency procedures and actions.

Therapy-Related Computers

Treatment planning system (TPS) capabilities have grown with computers and computing. Complex three-dimensional (3D) or four-dimensional (4D) image manipulation and dosage estimates may be available, depending on the TPS. Source calibration must utilize the same unit of activity as the TPS. SSG-46 cautions that such circumstances can cause serious errors. NRC Guideline 35 lists that testing should be done before TPS patient planning [9,17].

Source

The gamma-emitting radioactive materials used for the brachytherapy treatment are sealed inside capsules of certain shapes, sizes, and materials. In the context of HDR brachytherapy, these sealed radioactive materials are generally called sources, which have a cylindrical structure and are attached to a guidewire that facilitates the motion of the sealed material in the guide tube/applicator. This is unarguably the central component of the HDR brachytherapy procedure, and the manufacturing, possession, and use of these sources require immense regulatory compliance and trained individuals at work all over the chain of the source suppliers. The specific requirements are discussed below.

Conditions for Supplier of the Source

The source manufacturer and supplier, to make HDR brachytherapy source, has to meet certain requirements and standards given by international organizations. This includes the safety, sealing, design, and certification of the sources as required by the organizations.

Requirements for the Possession of Source

This section highlights the prerequisites for any individual to possess a source for medical use.

Requirements for the Use of Source

The most basic requirement for radioactive source use is to obtain permission from the competent authority of the state or country.

Calibration of Source

Calibration is nothing but the measurement of source strength and activity in the specified units. This is first done at the supplier end and then cross-checked by the user with necessary decay corrections.

Categorization of Source and Security Level

The various radioactive materials are classified primarily by IAEA guidelines into different categories according to the hazard they impose on the surrounding people and environment. Accordingly, the security level is suggested for the source/material, which defines the number of precautions to be taken during the delivery, use, and return of the source. Broadly, they are Category 2 Security Level B (demanding high security) sources [8-10].

Identification, Certification, and Licensing

The source supplied by the manufacturer and supplier, and possessed and used by the employer and licensee, must have a certificate specifying the radioactive material, its strength, and other necessary details. It shall be acquired along with a proper license for specified use.

Source Housing Integrity, Source Storage, and Security

The source housing is the part of the machine where the source is stored by default when not in use, whereas other storage containers are designed and used as needed and generally made of lead material. Both the container and housing have to meet certain design specifications. Source security refers to the security requirements that must be met at the organizational level.

Quality Assurance

QA is defined as the set of procedures required to be performed to ensure the safety of practice and establish the security of the professionals working at the facility.

Discussion

The mentioned reports, guidelines, and other auxiliary documents show that the formulation, commencement, and delivery of an HDR brachytherapy begins with the construction of a safe and secure layout for the installation and delivery of the procedure; then the choice, installation, and commissioning of the RAL unit, and finally the acquisition, installation, and commissioning of the source.

Layout

IAEA SSG 46 also recommends that radiation therapy institutions that use radioactive sources adopt technical measures apart from routine interlocks like closed circuit television (CCTV) cameras to detect malevolent activity. HDR brachytherapy and external beam radiation therapy should not share a shielded treatment room because it can slow down procedures. Electrical and mechanical systems should only be operated by authorized personnel who understand medical radiological equipment standards. As medical radiological equipment safety is critical, electrical and mechanical maintenance should be included in the quality assurance program and performed by the manufacturer or an authorized agent at a frequency recommended by the manufacturer [9]. NCRP Report 49 recommends visual inspection during construction to ensure specification compliance and identify faulty materials or workmanship that can be fixed more economically. Readers can see the detailed list in the report [13]. According to AERB Safety Code SC-1, radiation therapy installations should be assessed for flooding or fires and preventive measures applied. Consultation with firefighters and radiation therapy equipment manufacturers/suppliers to supply suitable firefighting systems should be done [16]. The AAPM report does not recommend combining the brachytherapy room with the operating room. It can complicate the interlock, shield, storage, and security of the afterloader, increase training of non-radiation oncology personnel, and create high-pressure treatment planning time constraints because anesthesia duration should be minimized and operating room time is costly [11].

Equipment

IAEA SSG-46 of the IAEA Safety Standards covers safety practices to prevent or reduce accidents. For RAL-type HDR units, the guideline's paragraphs 5.316-5.319 provide precise instructions. If the source gets stuck, one should keep a shielded container large enough for the largest applicator set next to the device [9]. All practical techniques are listed in SRS-47, and section 7.3 of the report details the procedures [10]. The AERB study emphasizes the employer/licensee's responsibility to create an emergency preparation plan, identify accountable parties, and outline emergency procedures for certain circumstances [15,16]. The NCRP Report 40 suggests standard radioactive cleanup procedures [12]. The NRC provides radiation emergency regulations [17].

Source

In addition to the above regulations, the IAEA SSG-46 recommends marking source storage facilities as radioactive and providing emergency contact information for the radiation safety officer (RSO), medical physicist, or other radiation safety professional [9]. NCRP Report 40 outlines source loss protocols. Any source loss must be notified to the radiation protection supervisor promptly. Prevention of acute local injury from direct contact with sealed sources requires all linen, dressings, clothing, equipment, and garbage containers to be held in a patient's cubicle until the radiation protection supervisor releases them or all sources are accounted for. In NCRP Report 40 appendix E, tables 6 and 7, and appendix B provide dosage estimates for such situations [12]. According to NCRP Report 182, any sealed source loss beyond an exempt quantity must be notified to the regulatory authority. Notification time depends on source category, licensing regulations, and regulatory agencies. Source theft can lead to regulatory action for failing to control radioactive material and serious health and safety issues for workers and the public [14].

Radioactive material leaks from sealed sources are usually caused by source containment damage. Regulated sealed sources must be leak-checked periodically to ensure containment. A typical sealed HDR brachytherapy source must withstand -40°C (20 minutes) to 600°C (1 hour), pressure of 25 kiloPascal (kPa) to 2 MegaPascal (MPa), and an impact of 50 g from 1 m. The leakage radiation dosage rate for unrestricted access and restricted access at 5 cm from the storage surface is 10 microGray/hour (µGy/h) and 100 µGy/h, respectively, and at 1 m from the source is 1 and 10 µGy/h [15,16]. AERB recommends building a physical protection system, which includes all systems and equipment that physically protect the radioactive source or site. These systems are designed on physical protection principles, which can be achieved through human actions and equipment. A good physical protection system combines human supervision and equipment. Vulnerability assessments are used in its design and evaluation [15,16]. According to AAPM, each facility must evaluate its on-site activity for source security and licensing needs. Due to the rapidity of dose delivery in HDR brachytherapy, the AAPM recommends that an authorized medical physicist (AMP) and authorized user (AU) be present for all patient treatments. The AMP must be immediately available throughout treatments; however, an emergency physician may substitute the AU. Radiation accidents are infrequent when radioactive sources are secure and safety measures during procedures are taken [11].

Future direction

As per the current international status regarding safety, various organizations are publishing guidelines, regulations, and codes for safety in HDR brachytherapy. This may become hectic for professionals to go through or opt for when it comes to applying safety standards at a given center. The current norm is to follow the guidelines established by the national regulatory authority and follow IAEA recommendations in the absence of the same. In the future, it can be of great ease if the international standards of safety come to a level of alignment with each other just like the dosimetric standards in radiotherapy. This will not only reduce the hassle of meeting different safety requirements but also will establish a common checkpoint for the correlation of standards and correction of wrong practices, if any.

## Conclusions

Radiation safety for patients, workers, and the public must be prioritized. Equipment safety and environmental preservation are also of equal importance. Facility management should be authorized, managed, and regulated according to the legal framework. Following protocols keeps occupational worker radiation doses under safe limits. IAEA provides a complete summary of regulatory requirements for radiation protection standards. Other organizations reference IAEA standards for their region/state/country. AERB regulates radiation facilities in India; therefore, most set-ups follow safety standards and instructions. As described by the standard organizations, the prime responsibility for maintaining radiation safety and compliance with radiation safety protocols is shared by the employer, licensee, the RSO, and working professionals. Meanwhile, the RSO in India is appointed by the hospital administration and is an individual who is an expert in matters of radiation safety and is licensed by the AERB of India. As defined by standard organizations, employers, licensees, RSO, and working professionals share the primary responsibility for radiation safety and protocol compliance. In India, the hospital's RSO is a radiation safety specialist nominated by the AERB, and the primary responsibility is attributed to the RSO.
